# Development and validation of virtual reality-based Rey Auditory Verbal Learning Test

**DOI:** 10.3389/fnagi.2022.980093

**Published:** 2022-09-14

**Authors:** Amihai Gottlieb, Glen M. Doniger, Shani Kimel-Naor, Oran Ben-Gal, Maya Cohen, Hila Iny, Michal Schnaider Beeri, Meir Plotnik

**Affiliations:** ^1^Center of Advanced Technologies in Rehabilitation, Sheba Medical Center, Ramat Gan, Israel; ^2^Joseph Sagol Neuroscience Center, Sheba Medical Center, Ramat Gan, Israel; ^3^Department of Psychiatry, The Icahn School of Medicine at Mount Sinai, New York, NY, United States; ^4^Department of Physiology and Pharmacology, Faculty of Medicine, Tel Aviv University, Tel Aviv, Israel; ^5^Sagol School of Neuroscience, Tel Aviv University, Tel Aviv, Israel

**Keywords:** memory and learning tests, neuropsychological tests, reliability and validity, Rey Auditory Verbal Learning test, validation study, virtual reality

## Abstract

**Objective:**

Translations and adaptations of traditional neuropsychological tests to virtual reality (VR) technology bear the potential to increase their ecological validity since the technology enables simulating everyday life conditions in a controlled manner. The current paper describes our translation of a commonly used neuropsychological test to VR, the Rey Auditory Verbal Learning Test (RAVLT). For this aim, we developed a VR adaptation of the RAVLT (VR-RAVLT) Which is based on a conversation with a secretary in a virtual office using a fully immersive VR system. To validate the VR-RAVLT, we tested its construct validity, its age-related discriminant validity and its test-retest validity in reference to the original gold standard RAVLT (GS-RAVLT).

**Method:**

Seventy-eight participants from different age groups performed the GS-RAVLT and the VR-RAVLT tests in a counterbalanced order in addition to other neuropsychological tests. Construct validity was validated using Pearson’s correlations coefficients and serial position effects; discriminant validity was validated using receiver operating characteristic area under the curve values and test-retest reliability was validated using intraclass correlation coefficients.

**Results:**

Comparing both RAVLTs’ format results indicates that the VR-RAVLT has comparable construct, discriminant and test–retest validities.

**Conclusion:**

the novel VR-RAVLT and the GS-RAVLT share similar psychometric properties suggesting that the two tests measure the same cognitive construct. This is an indication of the feasibility of adapting the RAVLT to the VR environment. Future developments will employ this approach for clinical diagnosis and treatment.

## Introduction

The term “ecological validity” in regard to neuropsychological tests refers to the relation of the performance on the tests and the performance in everyday life (Chaytor et al., 2006). Although it was assumed that traditional neuropsychological tests are able to tap the same executive functions that are used in day-to-day actions, there is evidence that this is not necessarily the case since poor performance on the tests was not ineludibly reflected in poor performance in everyday life (Wilson, 1993; Manchester et al., 2004; Sbordone, 2008; Bottari et al., 2009). This situation impairs the ability to rely on the results from traditional tests in order to intervene and appropriately support individuals with cognitive impairments.

Translations and adaptations of traditional neuropsychological tests to virtual reality (VR) technology bear the potential to increase their ecological validity since the technology enables simulating everyday life conditions in a controlled manner (Parsons et al., 2017; Gal et al., 2019; Plotnik et al., 2021). However, to date, attempts to translate neuropsychological tests to VR are still rare (Elkind et al., 2001; Pollak et al., 2009; Armstrong et al., 2013; Parsons et al., 2013).

Recently we reported on our successful study to translate the Color Trails Test, which is a cultural fair variant of the widely used Trail Making Test, to a VR environment (Plotnik et al., 2021). The current paper describes our study to translate another commonly used neuropsychological test to VR, the Rey Auditory Verbal Learning Test (RAVLT).

The RAVLT is a widely used verbal memory test (Rey, 1964; Vakil and Blachstein, 1997). Its main components consist of three parts: first, a list of 15-semantically unrelated items (list A) is read five times to the participants, whom are asked to recall as many items as they can remember after each time (trials 1–5). Then, a new interference list is introduced (list B) after which the participants are asked to repeat the original list (trial 6). In the third part the participants are asked to recall the original list (list A) after a 20–40 min interval (trial 7 – delayed recall). There is a large number of potential outcome measures that can be computed from the results of the RAVLT (Bowler, 2013) whereas we decided to use three common measures that were frequently mentioned in the literature (Geffen et al., 1990; Vakil and Blachstein, 1993; Guilmette and Rasile, 1995; Groth-Marnat, 2009; Vakil et al., 2010; Bowler, 2013; Vlahou et al., 2013): acquisition (ACQUISITION; sum of the scores on trials 1–5), retroactive interference (RI; trial 5 vs. the score in trial 6) and retention (RETENTION—trial 5 vs. the score on trial 7).

The RAVLT has shown good construct, test-retest and discriminant validity (Vakil and Blachstein, 1993; Tierney et al., 1996; Estévez-González et al., 2003; Schoenberg et al., 2006; Balthazar et al., 2010; Fichman et al., 2010; Ricci et al., 2012; Bowler, 2013) and high correlation with other neuropsychological tests such as Stroop (McMinn et al., 1988), Trail Making Test (Callahan and Johnstone, 1994), subscales of the Wechsler Adult Intelligence Scale-Revised (WAIS-R) (McMinn et al., 1988), and the Benton Visual Retention Test (de Sousa Magalhães et al., 2012). Additionally, the RAVLT’s results demonstrate robust serial positions effects of primacy and recency i.e., a tendency to better recall the first few and last few words in the lists (Bernard, 1991; Crockett et al., 1992).

As part of our overarching goal to create VR- based ecological valid adaptation of widely used neuropsychological tests (Plotnik et al., 2021), we created a VR adaptation of the RAVLT (VR-RAVLT). Our objective was to test its construct validity and its age-related discriminant validity in reference to the original gold standard RAVLT (GS-RAVLT) and its test-retest validity, among 78 participants from different age groups. We hypothesized that the tests would show that the new VR-RAVLT similarly evaluates verbal memory as the GS-RAVLT and has comparable, or even better, discriminant and test-retest validities.

## Materials and methods

### Creating the virtual reality adaptation of the Rey Auditory Verbal Learning Test

Virtual reality adaptation of the Rey Auditory Verbal Learning Test is designed to maintain the core features of the original GS-RAVLT (i.e., conserving the core task of reciting as many words out of a 15 words list read to the participant). As first stage, the current format goes beyond the “in clinic” evaluator reading lists of pseudo random words to the participant, by creating a typical real-life scenario during which the need to memorize words (i.e., places—see below) is intended to assist the participant in his/her daily function (i.e., the need to actually visit these places).

**FIGURE 1:**
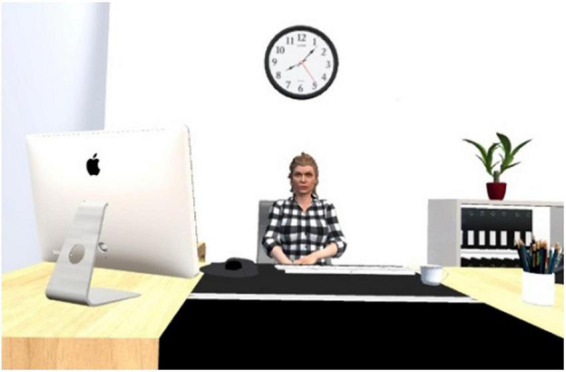
The virtual reality adaptation of the Rey Auditory Verbal Learning Test (VR-RAVLT). For RAVLT episodic, verbal memory test—recall of 15 items is tested five times sequentially (learning curve), once again after a new interference list (retroactive interference) and then again after 20 min (delayed recall). In the VR-adapted test, recall of the non-semantically related items is replaced by recall of real-life places-to-go dictated by a virtual personal assistant (avatar).

The VR-RAVLT places the participant in a virtual office with a virtual personal assistant (avatar) seated behind a desk. The avatar tells the participant a list of 15 places s/he needs to visit on the same day (see Supplementary Table 1), and that s/he must recall as many as possible. The avatar informs the participant that as she will be leaving early for the day, she will repeat the list to ensure the participant remembers all the places (i.e., similar to the procedure employed in the GS-RAVLT). List B consists of 15 places that the participant would need to visit on the next day. Participant responses are recorded by a research assistant in a form similar to what is used in the GS-RAVLT. Lists of places were matched for Hebrew word frequency using a 165-million word database (Linzen, 2009). Since the GS-RAVLT consists of alternate forms, two alternate lists were also created for the VR-RAVLT and tested in the study (see Supplementary Table 1). VR adaptation was performed in a full immersive VR system (HTC-Vive; New Taipei City, Taiwan). Figure 1 depicts the VR-RAVLT as viewed by the participant. Video demo is provided in the Supplementary material.

### Sample size justification

As we hypothesized that the VR-RAVLT would measure verbal memory similarly to the GS-RAVLT, we calculated the sample size based on bivariate normal model correlation with *r* ≥ 0.6 and alpha of 5% to achieve a power of 80% using Gpower (Erdfelder et al., 1996). It was determined that a total sample size of 15 participants was required. Therefore, we set a minimum of 15 participants in each of the study groups, i.e., young adults (YA), middle aged (MA), and older-adults (OLD).

### Participants

A total of 78 participants comprising of three cohorts of healthy participants were included: (1) YA; *n* = 29; (2) MA; *n* = 29; and (3) Cognitively normal OLD; *n* = 20. Table 1 depicts their demographic data. Inclusion criterion was “healthy men and women ages 18–90” while exclusion criterion was “Having any motor, balance, psychiatric, or cognitive impairment that may affect the ability to understand instructions or perform the tasks required.” Participants’ health status was confirmed by questioning. Two participants were excluded from the study as they failed to meet these criteria (one due to motor impairment and one due to a suspected cognitive impairment). The experimental protocol was approved by the local institutional review board (IRB). All participants signed a written informed consent prior to entering the study. The research was completed in accordance with Helsinki Declaration.

**TABLE 1:** Demographic data.

	Young adults (*n* = 29)	Middle aged adults (*n* = 29)	Older-adults (*n* = 20)	*P* (ANOVA/Chi square)
Age (mean ± SD, range)	24.1 ± 2.9 (19–29)	57.7 ± 4.0 (43–63)	71.8 ± 5.1 (66–86)	*p* < 0.0001
Gender (females, percentage)	17, 58.6%	21, 72.4%	13, 65.0%	n.s.
Education years (mean ± SD, range)	13.9 ± 2.0 (12–19)	16.8 ± 3.89 (12–30)	13.9 ± 3.0 (8–18)	*p* = 0.001

SD, standard deviation; ANOVA, analysis of variance; age was found to be significant different among all groups; the middle-aged group had significantly more education years as opposed to the young adults and the older-adults groups.

### Procedure

Participants performed GS-RAVLT and the VR-RAVLT test in a full counter-balanced order. The GS-RAVLT was performed according to its handbook instructions (Schmidt, 1996), i.e., by using two lists of 15-semantically unrelated items (two lists are also presented in the VR-RAVLT, recall the “Creating the virtual reality adaptation of the Rey Auditory Verbal Learning Test” section). In order to test the construct validity (concurrent validity) of the VR-RAVLT, the participants were also asked to perform four additional neuropsychological tests: the Montreal Cognitive Assessment (MOCA) (Nasreddine et al., 2005), the WAIS-R Digit Symbol Test (Wechsler and De Lemos, 1981), a Verbal Fluency Test (Lezak et al., 2004) and the WMS III digit span test (Wechsler, 1997). The MOCA is a highly valid and reliable, 10-min cognitive test that is widely used in numerous clinical and research setting and that taps a wide number of putative cognitive domains (Nasreddine et al., 2005). In the WAIS-R Digit Symbol Test the participants are required to draw as many symbols as they can according to a digit-symbol pairs index during 2 min and the test is considered to be sensitive to age, depression, brain damage and dementia (Wechsler and De Lemos, 1981). In the Verbal fluency test the participants are required to produce as many words as possible from specific categories in a time frame of 2 min and the test is considered as a valid method to detect cognitive impairment and dementia in clinical and research settings (Lezak et al., 2004). Finally, the forward and backward WMS III digit span test is part of the Wechsler Memory Scale (WMS) which requires the participants to repeat numbers in the same order as read aloud by the examiner, and in a reverse order and is considered as a valid measure of working memory which is one of the elements underlying general intelligence (Wechsler, 1997). The four tests were performed according to their handbook instructions and were done sequentially by all of the participants in between the performance of the two RAVLT formats. In order to examine the test-retest reliability, the GS-RAVLT and the VR-RAVLT were performed by the same participants also during a second visit, 2–3 weeks after the first one. As participants awaited the delayed recall component in both RAVLT test versions, they were asked to complete the additional pen and paper neuropsychological tests, which included removing and reapplying the VR goggles in the VR-RAVLT.

### Outcome measures and analyses

For the GS-RAVLT and the VR-RAVLT, the number of correct remembered items were recorded. Summary statistics (mean ± SD) were computed for each outcome measure (ACQUISITION, RI, and RETENTION). Before conducting the statistical analyses, we performed two *a priori* analyses. First, to verify suitability of parametric statistics, Shapiro–Wilk normality tests were run on the residuals that were calculated using analyses of variance (ANOVA) with a three-level independent group variable that were performed on each of the three outcome measures, per test. Of the six normality tests, five indicated non-normal distributions (Shapiro–Wilk statistic ≤0.91; *p* ≤ 0.007). Thus, we used non-parametric Kruskal–Wallis tests to assess effects of group (YA, MA, and OLD) within each test format and Wilcoxon signed rank tests to assess the effect of Format (GS vs. VR). Second, we tested the comparability of the two VR-RAVLT alternate forms by performing an independent samples Mann-Whitney test comparing both forms’ three main outcome measures. These comparisons reveled no significance results (*p* > 0.43) confirming the forms comparability and allowing to combine their results in the further analyses.

Construct validity (Concurrent validity) of the VR-RAVLT was evaluated by computing Pearson’s correlations coefficient between the GS-RAVLT and the VR-RAVLT tests’ outcome measures (ACQUISITION, RI, and RETENTION) and by comparing the Pearson’s correlations coefficients between each test’s outcome measures and the main outcome measures of the four additional neuropsychological tests (the MOCA test, the WAIS-R Digit Symbol Test, the Verbal Fluency Test, and the WMS III digit span test).

To calculate serial position effects, the participant’s recalled words for each test format were divided into three segments: Primacy (words 1–5), Middle (words 6–10), and Recency (words 11–15) in line with the literature (Bernard, 1991; Suhr, 2002). Then, these segments were summed and submitted to a repeated measures ANOVA to compare segment and test format effects.

Discriminant validity of the VR-RAVLT (i.e., ability to separate between the age groups) was evaluated by comparing the receiver operating characteristic (ROC) area under the curve values (AUC; range: 0–1, higher values reflect better discriminability) for the main outcome measures for each test format.

Test-retest reliability of the VR-RAVLT was validated by comparing the intraclass correlation coefficients [ICC; two-way mixed, effects, absolute agreement, (Koo and Li, 2016)] of the three outcome measures (ACQUISITION, RI, and RETENTION) computed from the results of the GS-RAVLT and the VR-RAVLT that were performed by the same participant cohorts at two visits, 2–3 weeks apart. By convention ICC >0.75 is considered good reliability (Koo and Li, 2016).

Level of statistical significance was set at 0.05. Statistical analyses were run using SPSS software (SPSS Ver. 24, IBM).

## Results

All of the participants complied with the VR-RAVLT platform, with no complains on any discomfort or inability to perform the instructions related to the verbal memory test.

### Main outcome measures

Table 2 presents the Summary statistics (mean ± SD) that were computed for each outcome measure.

**TABLE 2:** Summary statistics for each outcome measure – Overall and within the age groups.

			ACQUISITION (0–75)			RI (0–15)			RETENTION (0–15)		
VR-RAVLT (mean ± SD, range)			53.1 ± 8.8 (25–71)			1.0 ± 1.7 (−3 to 7)			0.08 ± 1.6 (−3 to 5)*		
YA	MA	OLD	57.3 ± 7.8 (33–71)^α^	54.0 ± 5.8 (38–64)^α^	45.8 ± 9.7 (25–61)^β^	0.5 ± 1.5 (−3 to 3)	1.2 ± 1.6 (−2 to 6)	1.4 ± 2.2 (−2 to 7)	0.5 ± 1.7 (-3 to 5)^α^	0.6 ± 1.3 (−2 to 5)^α^	1.5 ± 1.7 (−3 to 5)^β^
GS-RAVLT (mean ± SD, range)			54.3 ± 10.6 (26–71)			1.3 ± 2.1 (−2 to 8)			1.6 ± 2.5 (−3 to 10)*		
YA	MA	OLD	56.4 ± 8.1 (34–68)^α^	59.5 ± 7.2 (34–71)^α^	43.6 ± 10.4 (26–59)^β^	1.2 ± 2.4 (−2 to 8)	0.9 ± 1.6 (−1 to 6)	2.0 ± 2.0 (−1 to 6)	1.6 ± 2.6 (−3 to 9)^α^	0.4 ± 1.3 (−2 to 3)^α^	3.5 ± 2.8 (0–10)^β^

ACQUISITION: sum of trial 1-5; RI, Retroactive Interference, trial 5 – trial 7; RETENSION: trial 5 – Delayed Recall; α, β: *p* ≤ 0.01 (between age groups effect, within outcome measure); *p = 0.01 (between test version effect, overall).

Group effects were observed for the ACQUISITION and the RETENTION variables in the VR-RAVLT, H(2) = 19.49, *p* < 0.0001, H(2) = 8.31, *p* = 0.01, respectively with poorer scores for the older-adults group. A similar pattern of results was observed in the GS-RAVLT, H(2) = 26.13, *p* < 0.0001, H(2) = 15.44, *p* < 0.0001, respectively.

In regards to Format effects, only the RETENTION variable was found to be significant, *Z* = 2.49, *p* = 0.01, resulting from higher retention rates in the VR-RAVLT. None of the other test’s results were significant (*p* > 0.13).

Since the middle-aged group had significantly more education years in comparison to the young- and older-adults (see Table 1), we conducted a control analysis by omitting the participants with the highest years of education (≥20) from the MA group (*n* = 6) so that this parameter was no longer different between groups. We repeated the statistical analysis and found similar results (see details in section 1 of the Supplementary material).

### Construct validity

**FIGURE 2:**
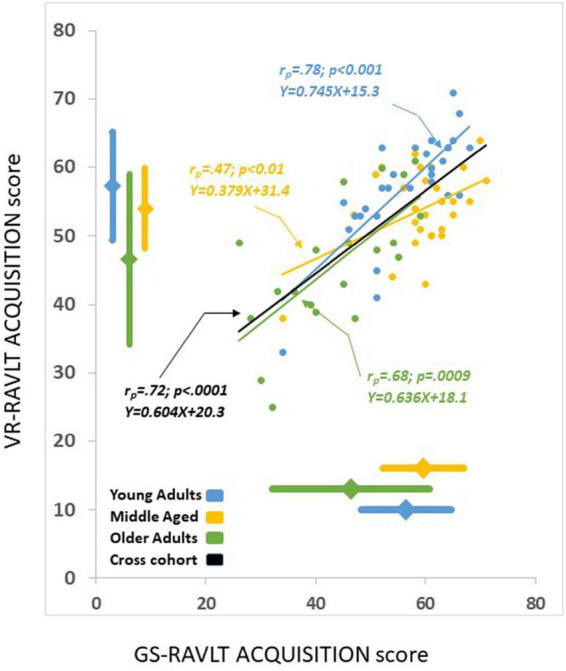
A visual depiction of the correlations between the gold standard-Rey Auditory Verbal Learning Test (GS-RAVLT) and the virtual reality adaptation of the Rey Auditory Verbal Learning Test (VR-RAVLT) acquisition scores, overall and within the young adults (YA), the middle aged (MA), and the older-adults (OLD) groups. Solid lines indicate linear fits. Diamonds and thick lines adjacent to the axes indicate averages and standard deviations. The correlations between the age groups ranged from 0.47 to 0.78; all correlations were significant. In order to determine whether the slopes of the regressions between the groups were homogeneous, we performed a univariate test and we found non-significant results F_(2,72)_ = 49.8, *p* = 0.68 indicating that the slopes are not significantly different.

**FIGURE 3:**
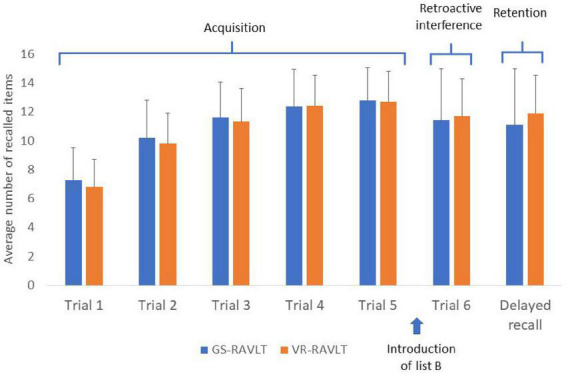
Average number of remembered items in each trial for both test formats. It can be appreciated that the Acquisition, Retroactive Interfere and the Retention patterns are similar.

Statistically significantly correlations were found between the GS-RAVLT and the VR-RAVLT for two of the three outcome measures (ACQUISITION: *r* = 0.721, *p* < 0.0001; RETENTION, *r* = 0.31, *p* = 0.005; RI, *r* = 0.18, *p* = 0.10; all 78 participants were included in this analysis, *Post hoc* power calculations are provided in section 2 of the Supplementary material, suggesting construct validity of the VR-RAVLT (see, e.g., Figure 2). To demonstrate these correlations, Figure 3 depicts the average number of remembered items on each trial for both test formats.

Table 3 presents the overall Pearson correlations coefficients between the RAVLT tests’ main outcome measures and the main outcome measures of other four neuropsychological tests.

**TABLE 3:** Pearson correlations between the RAVLT outcome measures and four other neuropsychological tests.

	MOCA score	WAIS-R digit symbol test score	WMS III digit span test Total	Verbal fluency test total
GS-RAVLT ACQUISITION	0.62**	0.57**	0.38**	0.24**
VR-RAVLT ACQUISITION	0.49**	0.54**	0.34**	0.28**
GS-RAVLT RI	−0.25*	−0.25*	–0.07	–0.13
VR-RAVLT RI	–0.09	–0.11	0.11	0.02
GS-RAVLT RETENTION	0.41**	0.30**	–0.05	0.14
VR-RAVLT RETENTION	0.29**	0.24*	0.14	0.23*

RI, retroactive Interference; YA, Young Adults; MA, Middle Aged; MOCA, Montreal Cognitive Assessment; WAIS-R, Wechsler Adult Intelligence Scale-Revised; WMS, Wechsler Memory Scale.

*p ≤ 0.05; **p < 0.01.

It can be appreciated that the overall correlation patterns between the neuropsychological tests and both formats of the RAVLT test are similar, also confirming the Construct validity of the VR-RAVLT. These correlations within each age group, scoring and Group effects related to the additional neuropsychological tests are reported in section 3 and (Tables 2, 3) of the Supplementary material.

**FIGURE 4:**
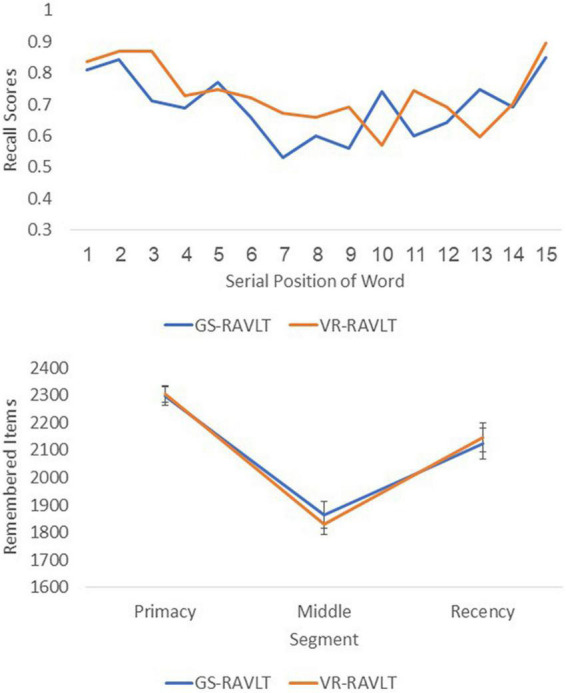
A visual depiction of the Serial Position as a function of the serial positions (top) and the primacy and recency segments (bottom) for the gold standard-Rey Auditory Verbal Learning Test (GS-RAVLT) and the virtual reality adaptation of the Rey Auditory Verbal Learning Test (VR-RAVLT). The recall scores and the remembered items are collapsed across participants and age groups.

Result of the serial position effect analysis yielded a significant main effect of segments (F_(2,12)_ = 7.1, *p* = 0.009, η2 = 0.54), but no main test format effect (F_(1,12)_ = 0.002, *p* = 0.97, η2 = 0.00) and no interaction effect (F_(2,12)_ = 0.08, *p* = 0.92, η2 = 0.01). These results portray robust primacy and recency effects that were similarly presented in both test formats, a pattern that also point to the VR-RAVLT’s good construct validity. Figure 4 depicts a visual impression of the serial position effects as a function of the serial positions and the primacy and recency segments.

### Discriminant validity

Table 4 depicts the ROC AUC values for discriminating between the three cohorts (bilateral discrimination). It can be appreciated that overall, the two formats have roughly similar discriminability, and consistent with the results presented in the “Main outcome measures” section (the source of the group effect are the values of the older-adults group). RI, however does not contribute to the ability to discriminate between the groups.

**TABLE 4:** AUC values from ROC curves.

	GS-RAVLT			VR-RAVLT		
	Acquisition	RI	Retention	Acquisition	RI	Retention
YA vs. MA	0.59	0.51	0.62	0.67*	0.62	0.52
YA vs. OLD	0.83**	0.63	0.72*	0.82**	0.61	0.71*
MA vs. OLD	0.91**	0.65	0.85**	0.76*	0.51	0.72*

AUC, area under the curve; ROC, receiver operating characteristic curves; YA, young adults; MA, middle aged adults; OLD, Older-adults; RI, retroactive Interference. Details on actual scores are not shown.

*p ≤ 0.05; **p < 0.0001.

### Test-retest reliability

For the VR-RAVLT (retest interval of 2–3 weeks), good reliability was yielded for the ACQUISITION (ICC = 0.732, *p* < 0.0001) and the RETENTION (ICC = 0.321, *p* = 0.050) and poor reliability was found for the RI (ICC = −0.185, *p* = 0.844) variable. For the GS-RAVLT a similar pattern was observed; good reliability was found for the ACQUISITION (ICC = 0.822, *p* < 0.0001) and the RETENTION (ICC = 0.669, *p* < 0.0001) variables, and poor reliability was found for the RI (ICC = −0.379, *p* = 0.992) variable.

## Discussion

The objective of the current work was to test the construct validity, the age-related discriminant validity and the test-retest validity of a novel, virtual reality format of the RAVLT, the VR-RAVLT, in reference to the original gold standard RAVLT. For this objective, seventy-eight healthy participants from three age groups performed both tests.

Overall, the results confirm construct validity of the VR-RAVLT, as reflected by significant correlations between the two formats in terms of the outcome measures constituting the main verbal memory performance (i.e., ACQUISITION, RETENTION). At odd was the retrospective interference—RI (see more below). Additionally, significant serial position effects (primacy and recency) were similarly demonstrated for both test formats, and comparable to those that were reported in the literature (Sullivan et al., 2002; Powell et al., 2004). Reviewing the correlations between the GS-RAVLT, the VR-RVLT and the other neuropsychological tests (Table 3), only the ACQUISITION and the RETENTION variables were highly correlated with other tests, which is also in agreement with the literature (McMinn et al., 1988; de Sousa Magalhães et al., 2012), while the RI seems to be less correlated. To the best of our knowledge, we are the first to report significant and high correlations between the MOCA scores, the Verbal Fluency Test scores and the WMS III digit span test scores and between the GS-RAVLT’s main outcome measures, further confirming its construct validity. In addition, similarly to the younger adults, among the older adults group we found high correlations between the VR-RVLT and the other neuropsychological tests which were comparable to the GS-RAVLT’s correlations with these tests (see Table 3 of the Supplementary material), suggesting that this group can understand, use, and be tested with the VR apparatus and indicating that this more technical and novel modality of cognitive assessment can be used for older adults.

Based on our samples, the RI measure does not seem to be similarly correlated with other neuropsychological tests’ outcomes and to be able to properly discriminate between healthy age cohorts, as the other RAVLT’s outcome measures. These results are in line with the few other cases in which this measure was calculated and reported [(Porter et al., 2003; Vakil et al., 2010; de Sousa Magalhães et al., 2012), but see Ricci et al. (2012) for a different result]. Considering these results, it seems that RI is not a sensitive index as the ACQUISITION or the RETENTION and its use in regards to measuring verbal memory should be re-visited.

In terms of discriminant validity, the VR-RAVLT was equivalent to the GS-RAVLT as being able to discriminate between older healthy adults and healthy young and middle-aged persons. When comparing the results to the literature, it should be noted that the RAVLT’s discriminant validity was mainly assessed by comparing clinical cohorts to healthy controls e.g., (Tierney et al., 1996; Estévez-González et al., 2003; Schoenberg et al., 2006; Balthazar et al., 2010; Ricci et al., 2012). At the same time, our AUC values are comparable to those obtained in studies that reported AUC (Ricci et al., 2012; Li et al., 2018). Notably, differentiating a clinical condition such as dementia or traumatic brain injury from normal cognition [i.e., (Schoenberg et al., 2006; Li et al., 2018)] is less difficult than differentiating between different age groups of cognitively normal individuals, as the cognitive changes related to aging are more subtle. Our results suggest that the RAVLT, including the VR-RAVLT, have the potential for discrimination of subtle early changes in cognition.

Finally, as to test-retest reliability, the VR-RAVLT yielded similar ICC values compared to the GS-RAVLT, and to those that were reported in the literature (Shapiro and Harrison, 1990; Vakil and Blachstein, 1997; Rezvanfard et al., 2011; de Sousa Magalhães et al., 2012; Nightingale et al., 2019).

The finding that the variance of both the VR-RAVLT and the GS-RAVLT ACQUISITION scores is greater among the OLD group than among the other groups (see Figure 2) could be attributed to some of the older participants having an incipient cognitive decline. In addition, variability in cognitive functioning increases with age (Glisky, 2007). We argue that, given the high correlation between the VR-RAVLT’s and the GS-RAVLT’s outcome measures found among this group, our results and conclusions are strengthened by these findings.

Re-framing the GS-RAVLT’s words remembering task as a “places-to-go” remembering task in the VR-RAVLT, generated a constraint of using 15 words that only describe places as the items to remember, as opposed to the 15 non-semantically related words that are used in the GS-RAVLT. This change may have increased the words semantic relatedness and thus may have caused a potential advantage in remembering them due to semantic priming (Foss, 1982) or by a better chance of using semantic encoding (Tulving, 1972). As a result, one possible outcome of our study was that the participants would score higher on the VR-RAVLT than the GS-RAVLT, an outcome that has not been observed. It is possible that the semantic distance of some of the places in the VR-RAVLT (e.g., coffee shop and airport) is similar to that of some words in the GS-RAVLT (e.g., house and river). An investigation of this hypothesis is warranted.

The current results do not demonstrate superiority of the VR-RAVLT over the GS-RAVLT in terms of age-related discriminant validity and test-retest validity, which may raise the question of the added value of the new test. In this context, the narrative (list of places to visit) and settings (an office) were designed to be ecological. However, the current task is still somewhat artificial i.e., to recall the same 15 item list seven times. This limitation was due to the requirement to translate the RAVLT to VR settings while not changing the task too much to keep the test’s core psychometric properties. On the other hand, our findings point to excellent construct validity of the VR format. Therefore, we posit that the current VR-RAVLT should evolve into more “real life” VR versions, while still maintaining the GS validity. This will provide additional benefits such as increased availability and usability by enabling to use them in various settings including patients’ homes. A next step will be to create a truly ecological verbal memory task which is more representative of daily tasks that would be valid and reliable while assessing verbal memory abilities, such as an everyday attempt to remember a grocery list or tasks. The combination of these advantages may make the VR version of the RAVLT more sensitive for the early ascertainment of incipient cognitive impairment.

As one of the limitations of the current study, the VR-RAVLT was not validated among non-healthy individuals such as those with cognitive impairment whereas one of RAVLT’s main purposes is to detect such impairment. Ongoing VR-RAVLT studies are recruiting cognitively impaired individuals.

In summary, our results suggest that the novel VR-RAVLT and the GS-RAVLT share similar psychometric properties (e.g., an increase in scores between trials 1 and 5, and then reduced scores in trials 6 and delayed). Coupled with the general high correlations between corresponding outcome measures and similar serial position effects, this suggests that the two tests measure the same cognitive construct (verbal memory). Taken together these results are an indication of the feasibility of adapting the RAVLT to the VR environment while preserving its core feature. This work, along with our recent work in the Trails Making Test (Plotnik et al., 2021) support that additional neuropsychological tests can be adapted to VR format as well. Finally, this work consists of one more milestone in the process of creating ecological valid versions of classic neuropsychological tests, which may yield clinical benefits by providing opportunities to understand neuropsychological functioning of patients in “close” to real life settings.

## Data availability statement

The raw data supporting the conclusions of this article will be made available by the authors, upon reasonable request.

## Ethics statement

The studies involving human participants were reviewed and approved by the Sheba Medical Center local IRB Committee. The patients/participants provided their written informed consent to participate in this study.

## Author contributions

GD, MB, and MP: conceptualization, supervision, project administration, and writing—review and editing. OB-G: software. SK-N, MC, and HI: investigation. AG: data curation, formal analysis, and writing—original draft. All authors contributed to the article and approved the submitted version.
