# Supplementary material for: Development and validation of virtual reality-based Rey Auditory Verbal Learning Test
Source: Front Aging Neurosci. 2022 Sep 14;14:980093. doi: 10.3389/fnagi.2022.980093 (PMC9519387; doi:10.3389/fnagi.2022.980093)
Supplement: Supplementary file 2 [file Data_Sheet_1.docx]

Supplementary Material

**Supplementary Table 1.** *The four different word lists places to visit used in the VR-RAVLT*

|  | **List A** | **List B** | **List C** | **List D** |
| --- | --- | --- | --- | --- |
| 1 | Coffee shop | Falafel stall | Shoemaker | IRS  (Internal reveniew service) |
| 2 | Electronics store | Hummus restaurant | Shawarma stall | Sewing workshop |
| 3 | Municipality | Bank | Market | Cellphone lab |
| 4 | Kindergarten | Swimming Pool | Accountant's office | Nursing Home |
| 5 | Bus station | Laundromat | Synagogue | University |
| 6 | Park | Eye doctor | Promenade | Parking lot |
| 7 | Sports center | Workplace | Greengrocer | Lawyer's office |
| 8 | Post-office | College | Clinic | Dentist |
| 9 | Bookstore | The mall | Hotel | Neighbor's house |
| 10 | Bakery | Supermarket | Museum | Gym |
| 11 | Public library | Soccer field | School | Culture Hall |
| 12 | Central bus station | Restaurant | Social security branch | Travel agent's office |
| 13 | Airport | Grandmother's house | Gas station | Hospital |
| 14 | Grocery | Cinema | Pharmacy | Barbershop |
| 15 | butcher | Garage | Pizzeria | Train station |

# Results of the control analysis

As described in the paper since the middle-aged group had significantly more education years in comparison to the young- and older-adults (see Table 1 in the main paper), we conducted a sensitivity control analysis by omitting the participants with the highest years of education (≥ 20) from the middle-aged group (n=6) so that this parameter was no longer different between groups.

Statistical analysis revealed that Group effects were observed for the ACQ and the RET variables in the VR-RAVLT, H(2) = 19.29, p < .0001, H(2) = 7.60, p = .022, respectively. The source of the effects is the differentiated ACQUISITION and RETENTION values exhibited by the older adult cohort. The exact same pattern of results was observed in the GS-RAVLT, H(2) = 25.21, p < .0001, H(2) = 12.99, p = .002, respectively.

In regards to Format effects, only the RETENTION variable was found to be significant, Z = 2.70, p= .007, resulting from a smaller retroactive interference in the VR-RAVLT. None of the other test's results was significant (p>.05).

These results are similar to what was found when all participants were included suggesting no bias due to different education levels.

# Post-hoc analysis to assess power of the construct validity correlation results.

Post-hoc power analysis of the correlations that were obtained in regards to the construct validity assessment of the VR-RAVLT, was calculated by submitting these correlations, an alpha of 5% and the sample size to the post-hoc power calculation module of Gpower (*Erdfelder, E., Faul, F., & Buchner, A. (1996). GPOWER: A general power analysis program. Behavior research methods, instruments, & computers, 28(1), 1-11.*‏). These calculations yielded that for ACQUISITION post-hoc power was 100%, for RETENTION 87% and for RI it was 47%. These results suggest that for the most the former measures, the study was adequately powered.

# Scoring and group effects in the additional neuropsychological tests

To verify suitability of parametric statistics, Shapiro–Wilk normality tests were run on the residuals that were calculated using analyses of variance (ANOVA) with a three-level independent group variable that were performed on each of the four neuropsychological tests outcome measures. Of the 4 normality tests, 3 indicated normal distributions (Shapiro–Wilk statistic ≥ 0.96; p ≥ 0.05). Thus, we used ANOVA tests to assess effects of Group (young, middle aged and elderly) within each test.

Results for the four ANOVA tests revealed that Group effects were observed only for the MOCA score and the WMS III digit span test total variables, F(2,77) = 5.93, p = .004, F(2,77) = 23.01, p < .0001, respectively. Bonferroni post-hoc analyses show that the sources of these effect are lower MOCA scored of the older-adults participants as compared to the other age groups (p<.01) and a difference between the digit span test scores among all age groups (p<.01).

**Supplementary Table 2.** *Scoring on the additional neuropsychological tests - Overall and within the age groups*

| **MOCA Score (mean ± SD, range)** | | | 26.5 ± 2.4 (20-30) | | |
| --- | --- | --- | --- | --- | --- |
| YA | MA | OLD | 25.0 ± 2.5 (20-30) | 26.9 ± 1.7 (23-30) | 27.2 ± 2.5 (21-30) |
| **WAIS-R Digit Symbol Test score (mean ± SD, range)** | | | 73.9 ± 16.8 (30-112) | | |
| YA | MA | OLD | 84.2 ± 13.9 (44-112) | 74.1 ± 12.1 (50 -101) | 57.7 ± 14.3 (30-89) |
| **WMS III digit span test Total** **(mean ± SD, range)** | | | 18.7 ± 4.2 (10-27) | | |
| YA | MA | OLD | 19.5 ± 4.1 (12-27) | 19.0 ± 3.7 (12-27) | 17.2 ± 4.8 (10-26) |
| **Verbal Fluency Test Total** **(mean ± SD, range)** | | | 44.8 ± 16.9 (12-143) | | |
| YA | MA | OLD | 48.4 ± 21.9 (18-143) | 43.5 ± 12.6 (12-66) | 41.4 ± 13.3 (23-69) |

**Supplementary Table 3.** *Pearson correlations between the RAVLT outcome measures and four other neuropsychological tests - within each age group*

|  | | | **MOCA Score** | | | **WAIS-R Digit Symbol Test score** | | | **WMS III digit span test Total** | | | **Verbal Fluency Test Total** | | |
| --- | --- | --- | --- | --- | --- | --- | --- | --- | --- | --- | --- | --- | --- | --- |
| **GS-RAVLT ACQUISITION** | | |  | | |  | | |  | | |  | | |
| **YA** | **MA** | **OLD** | .74** | .36 | .48* | .48** | .29 | .51* | .31 | .23 | .43 | .20 | .10 | .51* |
| **VR-RAVLT ACQUISITION** | | |  | | |  | | |  | | |  | | |
| **YA** | **MA** | **OLD** | .56** | -.06 | .44* | .51** | .13 | .30 | .23 | .23 | .34 | .22 | -.01 | .53* |
| **GS-RAVLT RI** | | |  | | |  | | |  | | |  | | |
| **YA** | **MA** | **OLD** | -.16 | -.13 | -.34 | -.43** | -.11 | .09 | -.03 | -.34 | .25 | -.14 | .04 | -.27 |
| **VR-RAVLT RI** | | |  | | |  | | |  | | |  | | |
| **YA** | **MA** | **OLD** | -.10 | -.13 | .10 | -.09 | -.32 | .42 | .00 | -.16 | .58** | .11 | -.16 | .25 |
| **GS-RAVLT RETENTION** | | |  | | |  | | |  | | |  | | |
| **YA** | **MA** | **OLD** | .25 | .21 | .49* | .34 | .15 | -.03 | .03 | .17 | -.22 | .18 | -.19 | .27 |
| **VR-RAVLT RETENTION** | | |  | | |  | | |  | | |  | | |
| **YA** | **MA** | **OLD** | .34 | .02 | .19 | .18 | .14 | .03 | .30 | .07 | -.16 | .22 | .27 | .12 |
